# Complex Insect–Pathogen Interactions in Tree Pandemics

**DOI:** 10.3389/fphys.2019.00550

**Published:** 2019-05-08

**Authors:** Alberto Santini, Andrea Battisti

**Affiliations:** ^1^Institute for Sustainable Plant Protection, National Research Centre, Sesto Fiorentino, Italy; ^2^Department of Agronomy, Food, Natural Resources, Animals and the Environment, University of Padua, Padua, Italy

**Keywords:** Dutch elm disease, cypress canker disease, pine wilt disease, vector, facilitator, antagonist

## Abstract

Tree pandemics are a major cause of economic and ecological loss in forest and urban ecosystems. They often depend on the introduction of a non-native pathogen, which is occupying the niche of a native, non-aggressive organism. Complex interactions with native insects carrying fungi and nematodes can be established based on the proximity of the aggressive pathogenic agents. Here we review three major pandemics of forest and urban trees in temperate ecosystems at world scale, i.e., the Dutch elm disease, the cypress canker, and the pine wilt disease. For each system, the relationships between aggressive and non-aggressive fungi and nematodes with the native insect vectors are presented. Hidden players such as insects, microorganisms or plants, which may have the role of facilitating or contrasting the performance of the agents, are also considered. Results suggest that pandemics rely on the introduction of a non-native pathogen that exploits well-developed interactions between native non-aggressive organisms and insects associated with trees. The success of the invaders depends on the morpho-physiological proximity of the players and on the mutual benefits resulting from the associations. Deciphering such interactions in native systems may help to predict the outcome of the introduction of new pathogens and the development of new tree pandemics.

## Introduction

When the Iron curtain collapsed at the end of the 1980s, probably none would have imagined that we were contributing to the last constitutive act of a new epoch, the Anthropocene (Crutzen and Stoermer, 2000; Crutzen, 2002; Lewis and Maslin, 2015). According to other authors (Rosenzweig, 2001), this was the start of the Homogocene, a term more related to invasion biology, which highlights the increasing rate of anthropogenic homogenization defined as the “gradual replacement of native biotas by locally expanding non-natives” (Olden et al., 2004). Plants and associated organisms have generally evolved in isolated assemblages, but when humans carry non-native pathogenic organisms into new environments, the latter may find suitable hosts lacking resistance genes and environments favoring their pathogenic behavior (Santini et al., 2018). This process may result in epidemics of newly emerging diseases, and, eventually, in a pandemic, i.e., a global epidemic. This process is greatly facilitated by the presence in the new environment of similar climatic conditions and of hosts phylogenetically related to those present in the native environment.

Pandemics may benefit from strict associations between plants and their herbivores and pathogens, which have developed through long periods of co-evolutionary change in species (Schluter, 2009). Such associations can occur even among very different taxa of microorganisms, arthropods, and plants (Biere and Tack, 2013). In some cases it is not just a dual interaction, but a very complex interaction among many organisms that shaped the genetic outcome of different species and the entire ecosystem. In some cases, the introduction of new pathogens is not followed by a disease epidemic until a new association with an insect vector is established. New associations between native insect vectors and non-native pathogens of trees resulted into a more efficient pathogen transmission, which are increasingly reported (Battisti et al., 1999; Brasier, 2001; Sousa et al., 2001; Lu et al., 2010; Luchi et al., 2012).

Aim of this review is to analyze factors that determined the success of three pandemics that affected trees, namely Dutch elm disease, cypress canker disease, and pine wilt disease (Table 1). All of them are well-known for their heavy economic, ecological, and landscape impacts. The common trait to the three pandemics is that a non-native pathogen has occupied the niche of a non-aggressive, native agent, replacing it almost completely in the invasion area. In addition, the relationships established by the native non-aggressive organism with a number of facilitating or antagonistic factors have been somewhat transferred to the non-native pathogens, with consequences that are difficult to predict.

**TABLE 1:** Organisms involved in three tree pandemics, i.e., Dutch elm disease, cypress canker, and pine wilt disease.

Host plant	Invasive pathogenic associated organism	Native non-pathogenic associated organism	Insect vector system	Facilitator/antagonist organism(s)
*Ulmus* spp.	*Ophiostoma novo-ulmi*	*Ophiostoma quercus*	*Scolytus* spp.	*Geosmithia* spp., *Proctolaelaps scolyti*, *Tarsonemus crassus*
*Cupressus* spp.	*Seiridium cardinale*	*Pestalotiopsis funerea*	*Phloeosinus* spp., *Orsillus maculatus*	*Megastigmus wachtli*
*Pinus* spp.	*Bursaphelenchus xylophilus*	*Bursaphelenchus mucronatus*	*Monochamus* spp.	Tree resistance and environmental conditions

## Case Study 1: Dutch elm Disease

Dutch elm disease is caused by some Ascomycete fungi of the genus *Ophiostoma* (Ophiostomatales, *Ophiostoma ulmi* s.l. for the sake of this review), and it is famous for being one of the most destructive diseases ever reported in the history of plant pathology. As a result of the disease, during the last century, adult elm trees were nearly wiped out from Europe, Asia, and North America. Two pandemics occurred, the first caused by *O. ulmi* started in Europe in the 1910s (Spierenburg, 1921) and rapidly spread in North America (Brasier, 2000; Guries, 2001). Around 1940, as most of the susceptible trees were killed, the effect of the disease diminished in Europe (Brasier, 1979). Just a few years later, in the 1950s, a second, and more destructive outbreak caused the death of elm trees in Europe, Western Asia, and North America (Brasier and Kirk, 2001), reducing many majestic trees to weedy shrubs (Mittempergher, 1989). This second, still active outbreak is due to a different species, the highly virulent *O. novo-ulmi* (Brasier, 1991), which has replaced *O. ulmi*. Moreover, two subspecies of *O. novo-ulmi* are known: *O. novo-ulmi* ssp. *novo-ulmi*, and *O. novo-ulmi* ssp. *americana* (Brasier and Kirk, 2001).

**FIGURE 1:**
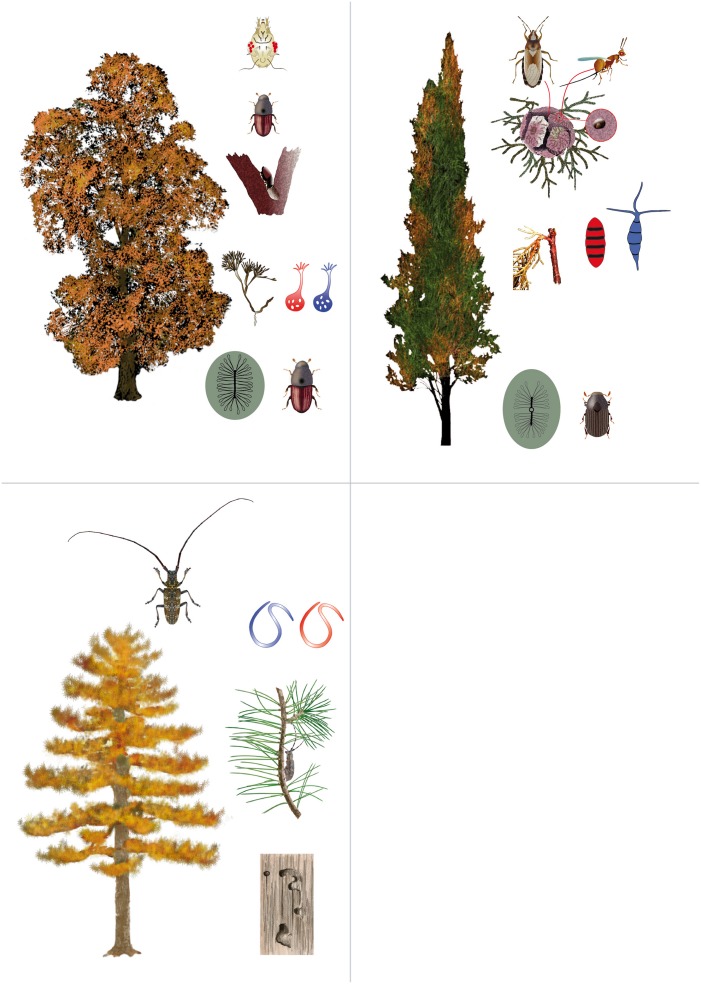
Agents involved in the development of tree pandemics causing dieback of elms (Dutch elm disease), cypress (cypress canker), and pines (pine wood nematode). The non-native, aggressive microorganism is in red color and the native, non-aggressive microorganism in blue color. Elm: elm bark beetles do both bark breeding on main stem and branches, and maturation feeding on twigs, through which they carry the indigenous saprotrophic fungus *Ophiostoma quercus* (blue) among trees, with the help of phoretic mites that carry the fungus in special parts of the body called mycangia. The introduction of the congeneric *O. ulmi* s.l. (red), a fungus having niche requirements similar to *O. quercus* but far more aggressive, caused the almost complete replacement of the endemic fungus. At the same time elm bark beetles and associated mites transport several species of *Geosmithia* (gray), which seems to have developed a mycoparasitic activity toward *O. ulmi* s.l. Cypress: cypress bark beetles do both bark breeding and maturation feeding on twigs, through which they carry the indigenous non-aggressive fungus *Pestalotiopsis funerea* (blue) among trees. The introduction of *Seiridium cardinale* (red), a fungus having niche requirements similar to *P. funerea* but far more aggressive, caused the development of lethal bark cankers. Both fungi, however, have another spreading pathway that involves seed cones. A fungus-infected cone can be inhabited by the nymphs of a true seed bug (*Orsillus maculatus*), the adults of which may carry a heavy spore load at emergence. Cones are infected when eggs are laid within the cone, most frequently via the emergence holes of a seed wasp (*Megastigmus wachtli*). Pine: pine sawyer beetles (*Monochamus* spp.) do both wood boring in stems and maturation feeding on twigs, through which they carry the nematodes of the genus *Bursaphelenchus* among trees. The association has evolved independently among native species of beetles and nematodes (in blue) in various parts of the world, and it is generally associated with mild and occasional symptoms on the host pines. The introduction of the congeneric *Bursaphelenchus xylophilus* (red) from North America to Asia and Europe, caused the development of large outbreaks of pine wilt disease. The invasive nematode impact depends on the susceptibility of the local host plant species and on the local environmental conditions.

The spread of this disease was particularly quick and effective because *O. ulmi* s.l. is supposed to have replaced the native non-aggressive species *O. quercus* in its ancient association with the elm bark beetles of the genus *Scolytus* in Europe (Brasier, 1990). *Ophiostoma quercus* is a fungus that colonizes a wide array of hardwood and conifer hosts, including elms (Taerum et al., 2018). Adult beetles contaminated with fungal spores emerge from pupal chambers in spring from the bark of dying infected elms and fly to feed at the crotches of young twigs of healthy elms. Infected beetles contaminate healthy elms by carrying the pathogen spores into the host’s vascular tissues. Spores germinate into a growing mycelium and reach the wide spring vessels, where the pathogen moves into a yeast multiplication phase (Webber and Brasier, 1984). Later, female beetles move to dying elms, mostly ought to the disease, to lay eggs under the bark of the stem and main branches, which is an ideal environment for both larval development (Rudinsky, 1962) and pathogen fructification (Webber and Brasier, 1984). Both asexual and sexual pathogen fruiting structures generate spores embedded in a sticky mucilage that facilitates their adhesion to the beetles. The new contaminated beetles emerge from the bark and move toward the crown of new healthy elms, completing the cycle (Figure 1).

Some other organisms are known for playing a less evident, but indeed important role in the dynamics of the beetle-fungi association. *Tarsonemus crassus* and *Proctolaelaps scolyti* are two mite species associated with *Scolytus* spp. that can carry *O. novo-ulmi* spores within their sporothecae or in the digestive system, respectively (Figure 1). They were described for significantly increasing the beetle’s spore spread efficiency (Moser et al., 2010).

Elm bark beetles also carry other ascomycetes such as *Geosmithia* spp., generally considered as saprotrophs or endophytes (Kolařík et al., 2008). In elms they were consistently isolated from beetles’ galleries, but never from dead wood or healthy trees (Pepori et al., 2015; Figure 1). A high frequency horizontal gene transfer of the cerato-ulmin gene between *O. novo-ulmi* and *Geosmithia* spp. has been recently reported (Bettini et al., 2014), suggesting that the two species do not just share the same habitat and vectors, but they show much closer relationships. A recent study (Pepori et al., 2018), providing direct and indirect evidence, supports the hypothesis that many *Geosmithia* isolates specific to elm recently turned to be mycoparasites of *O. novo-ulmi*. This may lead to the long term stabilization of population dynamics of various organisms involved in this complex disease.

## Case Study 2: Cypress Canker Disease

Cypress canker is caused by the fungal pathogen *Seiridium cardinale*, found first in California on Monterey cypress [*Hesperocyparis* (*Cupressus*) *macrocarpa*], which over a period of only a few years was completely destroyed in the plantations located in inland districts (Wagener, 1939). Monterey cypress, which is extremely susceptible and widely traded for ornamental purposes, had a major role in spreading the disease to other host species worldwide. In the course of the following decades, the disease was introduced through the trade of *H. macrocarpa* to Oceania, Europe, South America, and Africa, where it spread over various cypress species (Barthelet and Vinot, 1944; Grasso, 1951; Graniti, 1998; Della Rocca et al., 2013), with a tremendous impact. *Seiridium cardinale* is a wound pathogen and it occurs in the presence of small wounds due to abiotic or biotic agents (cold, hail, forced growth by fertilizers, insects, rodents) in the periderm of stems and branches through which the conidia or mycelium enter the inner bark (Danti et al., 2013).

Dispersal of the agent is little known in the area of origin, which has recently identified with California (Della Rocca et al., 2013). Wind is an unlikely vector because spores (conidia) are embedded in a sticky mucilage (Wagener, 1928, 1939; Panconesi and Ongaro, 1982). Insects are often assumed to be important carriers of the fungus, and some evidence for this has been obtained for bark beetles (Covassi et al., 1975) in the area of introduction in the Mediterranean region, especially when they moved from their breeding systems in an infected tree to a healthy one because of maturation feeding or new colonization attempts (Figure 1). As the fungus readily produces infectious spores in laboratory cultures when seeds are added to the substrate, and fruiting bodies are more abundant on cones than on the bark of infected trees (Intini and Panconesi, 1974; Battisti et al., 2000), cone and seed insects have been also considered. The seed bug *Orsillus maculatus* feeds on cypress seeds and inhabits the cone throughout its development. It shows a perfect overlap with the range of its major host, *Cupressus sempervirens*, in the Mediterranean region (Rouault et al., 2005). The cypress seed bug may feed on the seeds from outside the cone, penetrating through the cone scales with the mouthparts (adult), or from inside (nymph). However, the ovipositor cannot penetrate the cone scales, and therefore an incidental opening must be available for egg-laying to be possible (Figure 1).

Typically, such openings are provided when scales shrink and separate after colonization of the cone by the native, non-aggressive fungus (*Pestalotiopsis funerea*) that is introduced into the cone when the seed bug lays eggs into an emergence hole of the seed wasp *Megastigmus wachtli* (Roques and Raimbault, 1986). The seed wasp lays its eggs in the young cone and then emerges as an adult through a hole, thus creating a perfect way for the seed bug to access the inside of the cone for oviposition (Rouault et al., 2007). In an experiment performed in a cypress stand in northern Italy, it was shown that the fungus attack of the cones was related to insect feeding, and the seed bug appeared to be a major agent as traces of its feeding or oviposition were found in nearly all the cones killed by the fungi (Battisti et al., 1999). In addition, conidia are highly produced in fruiting bodies on the scales of infected cones and can be transported by rain or other occasional vectors to other parts of the tree, where they can eventually infect tissues (Figure 1).

The relationship between the seed bug *O. maculatus* and the fungi pathogenic to the cypress, depends ultimately upon the availability of holes suitable for egg laying in cones. Other cone and seed insects, especially the seed wasp *M. wachtlii*, produce such holes (Figure 1). In all likelihood, the relationship

evolved over a long period of coexistence between insects and the non-aggressive fungus *P. funerea*. However, the introduction of another more aggressive species, such as *S. cardinale*, with similar niche requirements may lead to dramatic consequences for the survival of the tree and the perpetuation of the whole system (Zocca et al., 2008).

## Case Study 3: Pine Wilt Disease

Pine wilt disease is caused by a species of nematode, *Bursaphelenchus xylophilus*, commonly known as pine wood nematode, and it is one of the most important forest diseases globally (Futai, 2013). It was first reported in the early 20th century and has in the last four decades devastated pine forests in eastern Asia and recently in Europe (Portugal and Spain), where the impacted area is expanding (Økland et al., 2010; Vicente et al., 2012; Naves et al., 2016). The most likely origin of the different introductions is North America (Li et al., 2018; Mallez et al., 2018).

The disease is characterized by systemic wilting symptoms derived from sudden interruption of water transport in stem tissues (Fukuda, 1997), induced by *B. xylophilus* (Figure 1). In its native range, the nematode is vectored by native North American longhorn beetle species, *Monochamus carolinensis*, *M. mutator*, *M. scutellatus*, *M. titillator* (Coleoptera: Cerambycidae), and in Asia and Europe by congeneric, native longhorn beetles, *M. alternatus* and *M. galloprovincialis*, respectively. The possibility for *B. xylophilus* to switch among vector species in very different geographic areas is the key to understand its success and spread, which are mainly related to the beetle activity (flight) and human-assisted passive (with timber) dispersal (de la Fuente et al., 2018).

Colonization of beetles by the nematodes happens in the insect pupation galleries created in the wood (Figure 1). Juvenile nematodes in the dispersal phase are attracted by chemical signals released by the pupae (Zhao et al., 2013) and enter the tracheal system of young adult beetles. After emergence, beetles mature by feeding on the bark of young twigs of healthy host trees. The nematodes then leave the insect and enter the host tree through the feeding wounds. This is referred to as the primary infection, after which the nematodes molt to adult stage, start propagation, and spread throughout the tree to cause wilting symptoms. A secondary infection is possible, and it is associated with the oviposition into the bark of weak or dying trees (Kobayashi et al., 1984). A precise mechanism of pathogenicity has not been clarified yet, although it has been suggested that a hypersensitive reaction occurs in susceptible pine tissue, involving water transport and sudden appearance of disease symptoms (Fukuda, 1997). Such a reaction, however, is not always immediate and the nematode can survive asymptomatically in pine tissues until conducive conditions are established (Takeuchi, 2008).

The pine wilt disease caused by *B. xylophilus* and native *Monochamus* in North America is sporadically causing severe symptoms and damage. Similar results have been observed with combinations of congeneric native nematodes and vectors in Asia and Europe. *Bursaphelenchus mucronatus* is a phylogenetically sister species of *B. xylophilus* in Asia and Europe, where it occurs with two subspecies in each region (Kanzaki and Giblin-Davis, 2018). In all native nematode-native vector associations, the symptoms appear only when trees are challenged by strongly adverse conditions such as heavy environmental stresses such as abnormal water, light, and temperature conditions (Kanzaki and Giblin-Davis, 2018). In the introduction range of *B. xylophilus*, however, typical wilting symptoms on susceptible pine species may appear also when environmental conditions are not that stressful. To cause tissue and systemic symptoms, nematodes must in any case overcome host resistance, which is based on a complex biochemical interaction between the nematode and the host, where the insect vector does not seem to play an important role. The response of the tree to the nematode has been analyzed and a few resistance-related genes have been identified, especially in resistant species that show only weak reactions (Hirao et al., 2012). This suggests that nematodes do not propagate or disperse widely within the tree even if the nematode succeeds during the primary infection.

## Discussion and Conclusion

The three pandemics analyzed here show that non-native pathogens have efficiently replaced native non-aggressive organisms and caused a significant impact. The mechanisms through which this had happened, however, differ substantially among the three case studies.

In the pine wilt disease, both nematodes and vectors are taxonomically close. The non-aggressive and aggressive nematodes belong to the same genus as well as the native and non-native pine sawyers (Cesari et al., 2005; Kanzaki and Giblin-Davis, 2018). A different situation is observed for Dutch elm disease and cypress canker, as in both cases there is limited information about the nature of the vectors in the native range of the aggressive fungus. Non-aggressive and aggressive fungi, however, are taxonomically close in both diseases. Even if the origin of *O. ulmi* s.l. is still unknown, it is strictly related to *O. quercus* (Brasier, 1990; Taerum et al., 2018) and this fact has greatly facilitated replacement of the latter. It has been hypothesized that repeated hybridization of the two species may have occurred, with introgression in *O. ulmi* of useful characters such as vegetative compatibility genes from *O. quercus*, eventually resulting in genetic swamping (Brasier, 2001). In cypress canker, the fungi are members of the same family, i.e., Pestalotiopsidaceae (Senanayake et al., 2015).

Affinity of the ecological niche occupied by the fungi and nematodes in native and non-native areas is a trait shared in the three case studies and it seems to be more important than taxonomic relatedness. For the pine wood nematode, the ecological niche is virtually the same, with possible differences due to the host plant species and related morphology, which could be more or less permissive to the nematode invasion in the tissues (Futai, 2013). The niche of non-aggressive fungi associated with elms and cypresses matches perfectly with that of the invasive pathogens. In addition, all the vectors considered here have a limited specialization and high adaptation potential, so that a newcomer can easily find a way to be transported and the fitness of the new association is maximized. In addition, the non-native pathogens display a higher Darwinian fitness that increases after episodic selection episodes such as hybridization with native species and gene introgression (Brasier, 1995), facilitating the complete replacement of the native species (Brasier, 2001).

The key for success of the invasion and development of the pandemic relies on the increase in fitness of arthropod vectors. In all three systems, the arrival of the aggressive pathogen resulted in larger amounts of breeding material for the vectors and in the consequent spread and population growth. This is, however, a transient condition at least in a local scale, as long as the host plants die and cannot support more growth. On a larger scale, however, it could be favorable to vectors capable of long-distance dispersal, as it seems to be in the three systems (Rouault et al., 2005; Javal et al., 2018; Kanzaki and Giblin-Davis, 2018). In that case, the disease may wax and wane in space and time as long as the host plant regenerates and become suitable to colonization. This is the typical case of the Dutch elm disease dynamics after the introduction of the aggressive fungus (Brasier et al., 2004). Reconciling the dynamics of diseases over sub-epidemic level is possible through (i) changes in the genetics of the players, such as in the pine wood nematode (Li et al., 2018), (ii) the antagonistic role of some organisms, such as the *Geosmithia* competitor of *O. novo-ulmi* (Pepori et al., 2018) and the egg parasitoids of the seed bug (Rouault et al., 2007), and iii. a decreasing demographic plasticity of the pathogen (Garbelotto et al., 2015).

Humans may become leading forces in shaping new associations (Rosenzweig, 2001; Olden et al., 2004), especially when action is taken to modify the responses of the local native communities to the invader. In all three case studies, tree plantations are clearly more exposed than natural forests to pandemics, as shown for a number of other pests and pathogens (Wingfield et al., 2015). Unraveling the relationships that result from the accidental introduction of a non-native pathogen with the local communities of host trees and associated organisms is a fundamental challenge for future research on the ecology of forest ecosystems worldwide. New models and tools are required to address these challenges, such as species distribution models that are commonly used by governmental institutions for planning surveillance programs and decide where to concentrate efforts and resources (Lantschner et al., 2018; Poland and Rassati, 2019). The new associations in the invaded environment can completely alter the predictions for establishment and spread of certain non-native pathogens and may results in pandemics with tremendous consequences on human economy and ecosystems.

## Author Contributions

AS and AB conceived and wrote the manuscript.

## Conflict of Interest Statement

The authors declare that the research was conducted in the absence of any commercial or financial relationships that could be construed as a potential conflict of interest.
